# Effects of heroin self-administration and forced withdrawal on the expression of genes related to the mTOR network in the basolateral complex of the amygdala of male Lewis rats

**DOI:** 10.1007/s00213-022-06144-2

**Published:** 2022-04-25

**Authors:** Marcos Ucha, David Roura-Martínez, Raquel Santos-Toscano, Roberto Capellán, Emilio Ambrosio, Alejandro Higuera-Matas

**Affiliations:** 1 Department of Psychobiology, School of Psychology, National University for Distance Learning (UNED), C/ Juan del Rosal 10, 28040 Madrid, Spain; 2 Centre National de La Recherche ScientifiqueInstitut de Neurosciences de La Timone, UMR 7289, Aix Marseille Université, Marseille, France; 3 School of Pharmacy and Biomedical Sciences, University of Central Lancashire, Preston, UK

**Keywords:** mTOR, Rictor, Heroin, Basolateral complex of the amygdala, Incubation of seeking

## Abstract

**Rationale:**

The development of substance use disorders involves long-lasting adaptations in specific brain areas that result in an elevated risk of relapse. Some of these adaptations are regulated by the mTOR network, a signalling system that integrates extracellular and intracellular stimuli and modulates several processes related to plasticity. While the role of the mTOR network in cocaine- and alcohol-related disorders is well established, little is known about its participation in opiate use disorders.

**Objectives:**

To use a heroin self-administration and a withdrawal protocol that induce incubation of heroin-seeking in male rats and study the associated effects on the expression of several genes related to the mTOR system and, in the specific case of Rictor, its respective translated protein and phosphorylation.

**Results:**

We found that heroin self-administration elicited an increase in the expression of the genes *Igf1r*, *Igf2r*, *Akt2* and *Gsk3a* in the basolateral complex of the amygdala, which was not as evident at 30 days of withdrawal. We also found an increase in the expression of Rictor (a protein of the mTOR complex 2) after heroin self-administration compared to the saline group, which was occluded at the 30-day withdrawal period. The activation levels of Rictor, measured by the phosphorylation rate, were also reduced after heroin self-administration, an effect that seemed more apparent in the protracted withdrawal group.

**Conclusions:**

These results suggest that heroin self-administration under extended access conditions modifies the expression profile of activators and components of the mTOR complexes and show a putative irresponsive mTOR complex 2 after withdrawal from heroin use.

## Introduction

The development of compulsive drug use and the elevated risk of relapse even after protracted abstinence periods are two cardinal aspects of substance use disorders that involve synaptic plasticity processes (Kalivas and O’Brien 2008; Kasanetz et al. 2010; Dong et al. 2017). Among the countless mechanisms involved, the mechanistic target of rapamycin kinase (mTOR) plays a central role in plasticity processes (Costa-Mattioli et al. 2009; Stoica et al. 2011; Liu-Yesucevitz et al. 2011). mTOR is a serin/threonine kinase that interacts with other proteins to form two functional complexes: the mTOR complex 1 (mTORC1) and the mTOR complex 2 (mTORC2). Both complexes share several components, such as mTOR itself, the mammalian lethal with Sec13protein8 (mLST8) (Kim et al. 2003), DEP containing mTOR interacting protein (Deptor) (Peterson et al. 2009) and the Tti1/Tel2 complex (Kaizuka et al. 2010). In addition, the mTORC1 also includes the regulatory-associated protein of mTOR (Raptor) (Hara et al. 2002) and the proline-rich Akt substrate 40 kDa (PRAS40) (Haar et al. 2007; Sancak et al. 2007; Thedieck et al. 2007), while the specific components of the mTORC2 are the rapamycin-insensitive companion of mTOR (Rictor) (Sarbassov et al. 2004; Jacinto et al. 2004), the mammalian stress-activated map kinase interacting protein 1 (mSin1) (Frias et al. 2006; Jacinto et al. 2006) and the proteins observed with Rictor 1 and 2 (Protor1/2) (Pearce et al. 2007; Thedieck et al. 2007) (see Fig. 6).

Among the diverse processes regulated by both complexes, mTORC1 modulates protein, lipid and nucleotide synthesis (Porstmann et al. 2008; Ma and Blenis 2009; Düvel et al. 2010), autophagy (Blommaart et al. 1995), mitochondrial metabolism (Schieke et al. 2006; Cunningham et al. 2007) and cytoskeletal organisation (Choi et al. 2002), while mTORC2 is involved in the regulation of ion transport (García-Martínez and Alessi 2008) and actin cytoskeleton remodelling (Sarbassov et al. 2004). In addition, mTORC2, through the control of Akt function (Sarbassov et al. 2005), modulates cellular processes such as metabolism, survival, apoptosis, growth and proliferation (Madhunapantula et al. 2011). Interestingly, mTORC2 can regulate mTORC1 activity acting through Akt (Sarbassov et al. 2005), and, conversely, mTORC1 modulates mTORC2 through p70S6K (Julien et al. 2010), adding complexity to the regulation of the network**.** The mTOR network responds to a wide variety of factors, ranging from metabolic variables like oxygen, glucose or amino acid levels (Arsham et al. 2003; Shaw et al. 2004; Inoki et al. 2006; Sancak et al. 2008) to growth factors (Inoki et al. 2002; Takei 2004) or neurotransmitter signalling (Polakiewicz et al. 1998; Perkinton et al. 1999, 2002; Beaulieu et al. 2004, 2007; Santini et al. 2009; Meffre et al. 2012). In addition, there is evidence of modulation of this network by drugs of abuse (Muller and Unterwald 2004; Zhang et al. 2007; Perrine et al. 2008; Neasta et al. 2010), of its involvement in several reward mechanisms that mediate addiction-related phenomena, and also about the therapeutic potential of the pharmacologic manipulation of this signalling cascade (for a review about this exciting field, see Ucha et al. (2020).

Although the relationship between drug addiction and the mTOR network has been characterised to a great extent, most of the studies have focused on psychostimulant use disorder (Dayas et al. 2012; Neasta et al. 2014). Surprisingly, although opioids have been in the spotlight due to the recent opioid epidemic in the USA (Volkow et al. 2014; Kolodny et al. 2015; Cerdá et al. 2021; Ciccarone 2021), research about the relationship between opioid use disorders and mTOR related signalling is scarce (Ucha et al., (2020)). To fill this gap in the literature, in a previous study, we examined the effects of morphine self-administration and its extinction on the gene expression and protein phosphorylation of different elements of the mTOR network. We observed an increase in expression of one component of mTORC1, Raptor (regulatory-associated protein of mTOR), and one of its effectors, EIF4EBP2 (eucaryotic translation initiation factor 4E-binding protein 2), in the basolateral amygdala (BLA) after self-administration (Ucha et al. 2019). Previously, Cui and co-workers had also shown that the acquisition of morphine conditioned place preference (CPP) was correlated with the activation of the mTOR pathway in the CA3 hippocampal region, and that the blockade of the pathway with rapamycin, an mTOR inhibitor, impaired CPP (Cui et al. 2010). In addition, the development of opiate CPP is mediated by a reduction in insulin receptor substrate-2 (IRS2)/Akt and mTORC2 activity in the VTA, which elicits a decrease in the size of dopaminergic ventral tegmental area (VTA) neurons and their dopamine release to the nucleus accumbens (NAcc) (Russo et al. 2007; Mazei-Robison et al. 2011).

The mTOR network also seems to be involved in the relapse to opioid consumption. For example, a single dose of rapamycin was able to reduce the craving elicited by drug-related cues in persons with a heroin use disorder (Shi et al. 2009). In rodents, there is a reduction in the levels of phosphorylated Akt (a protein involved in the dynamics of both mTOR complexes) in the prefrontal cortex (PFC) and the ventral hippocampus after morphine CPP reinstatement. Moreover, unilateral administration of SC79, an Akt activator in the medial prefrontal cortex (mPFC) and the contralateral ventral hippocampus prevented CPP reinstatement (Wang et al. 2018). Apart from this, rapamycin was able to block the reconsolidation of morphine, cocaine and alcohol CPP in a dose-dependent fashion, suggesting that the mTOR network could be a potential therapeutic target for substance use disorders (Lin et al. 2014).

In this study, we aimed to assess the possible relationship between the mTOR network and opioid consumption and risk for relapse. For this purpose, we submitted rats to a heroin self-administration protocol under extended access conditions, a paradigm that induces escalation of drug intake and increased motivation for consumption (Ahmed et al. 2000). Subsequently, we used a model of incubation of heroin-seeking, based on the gradual increase (1 day of withdrawal versus 30 days) in reactivity to drug-associated cues that seems to be one of the causes underlying relapse risk. This phenomenon, common to humans and rodents, has been observed for heroin (Shalev et al. 2001; Nava et al. 2006; Roura-Martínez et al. 2020b), cocaine (Grimm et al. 2001; Parvaz et al. 2016; Roura-Martínez et al. 2020b), nicotine (Bedi et al. 2011; Markou et al. 2018), alcohol (Bienkowski et al. 2004; Li et al. 2015) methamphetamine (Shepard et al. 2004; Wang et al. 2013) as well as non-drug rewards as sucrose (Grimm et al. 2002; Roura-Martínez et al. 2020b) or palatable food (Krasnova et al. 2014) (see Venniro et al., (2021) for a recent review). We analysed the expression of three genes coding for receptors related to the mTOR network (*Igf1r*, *Igf2r* and *Insr*), six genes coding upstream second messengers of the network (*Akt1*, *Akt2*, *Gsk3*, *Gsk3b*, *Pdk1* and *Pi3ca*), three coding components mTOR complexes (*mTOR*, *Rptor* and *Rictor*) and three genes coding for downstream mediators and effectors (*Rps6kb1*, *Rps6* and *Eif4ebp2*). Given that we found an increase in the expression of Rictor, a regulator protein specific to mTORC2, and that the increased activity of this protein could contribute to explaining some of the results obtained, we decided to analyse the total levels of this protein and its phosphorylation at the threonine 1135 residue, an essential regulator of mTORC2 recruitment.

In our previous study using morphine self-administration and extinction, we only found significant effects of the treatment in the BLA (Ucha et al. 2019). In addition, the BLA has a key role in the formation of drug-related memories and the encoding of their emotional value (for a review see Luo et al., 2013), and also seems to be involved in heroin-seeking restatement (Fuchs and See 2002). For these reasons, we focused our research on the basolateral complex of the amygdala -BCA- (which encompasses the BLA).

## Experimental procedures

### Animals

Male rats of the Lewis strain were used (Harlan International Ibérica, *n* = 48), between 300 and 320 g of weight at the beginning of the experiments. The animals were kept in the vivarium in a light–dark cycle (on at 08:00 am), at a constant temperature (20 ± 2 °C) and relative humidity (50% ± 10%), with ad libitum access to water and food (standard commercial rodent diet A04/A03: Panlab). Since their arrival, the animals were housed in groups of three until catheterisation surgery, 1 week before the start of the self-administration sessions. Subsequently, they were single-housed to prevent potential damage to the catheters. All the animals were maintained and handled according to European Union guidelines for the care of laboratory animals (EU Directive 2010/63/EU governing animal experimentation), and the Ethical Committee of UNED approved all the experimental procedures. With the aim of complying with the reduction principle of the 3Rs, these animals belong to a subset of the rats used in our previous experiments (Roura-Martínez et al. 2020a, b).

### Experimental groups

Animals were initially preassigned to vehicle or heroin self-administration groups. After the self-administration phase, the following groups were matched for the consumption of either heroin or saline: heroin withdrawal 1 day (HW1, *n* = 8), vehicle withdrawal 1 day (VhW1, *n* = 8), heroin withdrawal 30 days (HW30, n = 8), vehicle withdrawal 30 days (VhW30, *n* = 8), heroin withdrawal 1 day seeking test (HTest1, *n* = 8) and heroin withdrawal seeking test (HTest30, *n* = 8). The rats of the HW1, VhW1, HW30 and Vh30 groups were only used for tissue harvest and molecular analysis and did not undergo extinction tests, while the HTest1 and HTest30 groups were only used for the behavioural tests of incubation of heroin-seeking*.*

### Surgery

All the animals were submitted to intravenous catheterisation surgery. Rats were anaesthetized with an isoflurane/oxygen mixture (5% isoflurane during induction; 2% ± 0.5% for maintenance), and a polyvinyl chloride catheter (0,16 mm i.d.) was inserted into the right jugular vein of the animal approximately at the level of the atrium and secured there with surgical thread. The catheter was fixed subcutaneously around the neck, exiting the skin at the midscapular region. A pedestal of dental cement was then mounted on the skull of the rat to attach the tethering system. After surgery, the rats were allowed to recover for 7 days and an NSAID was added to the drinking water (meloxicam—Metacam™: 15 drops of a 1.5 g/ml solution per 500 ml of water). Until the end of the self-administration procedure, the catheters were flushed daily with a sterile saline solution containing sodium heparin (100 IU/ml) and gentamicin (1 mg/ml) to maintain catheter patency and to prevent infections.

### Apparatus

Twelve operant conditioning chambers (l = 300 mm; w = 245 mm; h = 328 mm) (Coulborne Instruments), each equipped with a microliter injection pump, were used for the heroin self-administration and seeking tests. A catheter was connected to the rat and held in place with a spring-tether system, and a rotating swivel, which allowed the animals to move freely inside the chamber. Two levers placed 14 cm apart were available throughout all the sessions, one of them inactive.

### Self-administration

A week after recovering from surgery, the rats underwent 10 daily sessions of either heroin or vehicle self-administration. During the light phase of the light cycle, for 6 h rats were allowed daily access to heroin (0.075 mg/kg in a sterile saline − 0.9% NaCl − solution) or its vehicle alone under a fixed-ratio 1 reinforced schedule. The house light was off during the sessions, although we allowed some environmental light to respect the light/dark cycle of the animals. During these sessions, one active lever press resulted in a heroin infusion (100 μl delivered over 5 s) and a cue light was switched on for 10 s. Each active lever press was followed by a 40-s time-out during which the responses had no effects but were still registered. In the first two self-administration sessions, two sucrose pellets were placed on the active lever to facilitate the acquisition of self-administration behaviour.

### Seeking tests

The rats of the HTest1 and HTest30 groups underwent seeking tests 1 day or 1 month after the last self-administration session. These tests were performed under the same conditions as the self-administration sessions, but the active lever presses were not followed by an infusion, and the duration of the sessions was 3 h. During the withdrawal period, the animals were still handled daily but their catheters were no longer flushed with the gentamicin/heparin solution.

### Tissue collection and processing

**Fig. 1:**
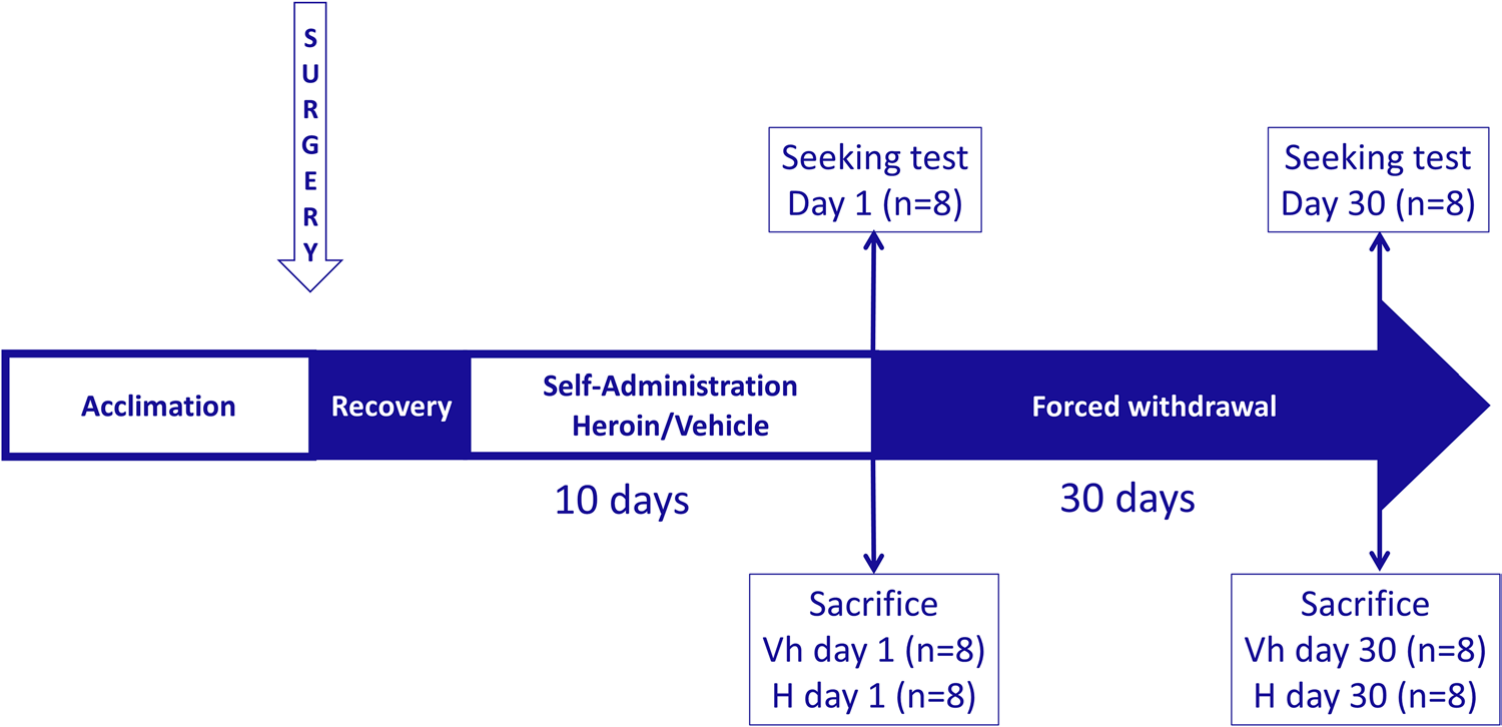
Experimental schedule of the study

Rats of the HW1, VhW1, HW30 and Vh30 groups were weighed and euthanised by decapitation 1 day or 1 month after the last self-administration session, between 11:00 and 13:00 a.m. The rat’s brain was extracted and submerged in isopentane chilled on dry ice for ten seconds and stored at − 70 °C. (Fig. 1)

**Fig. 2:**
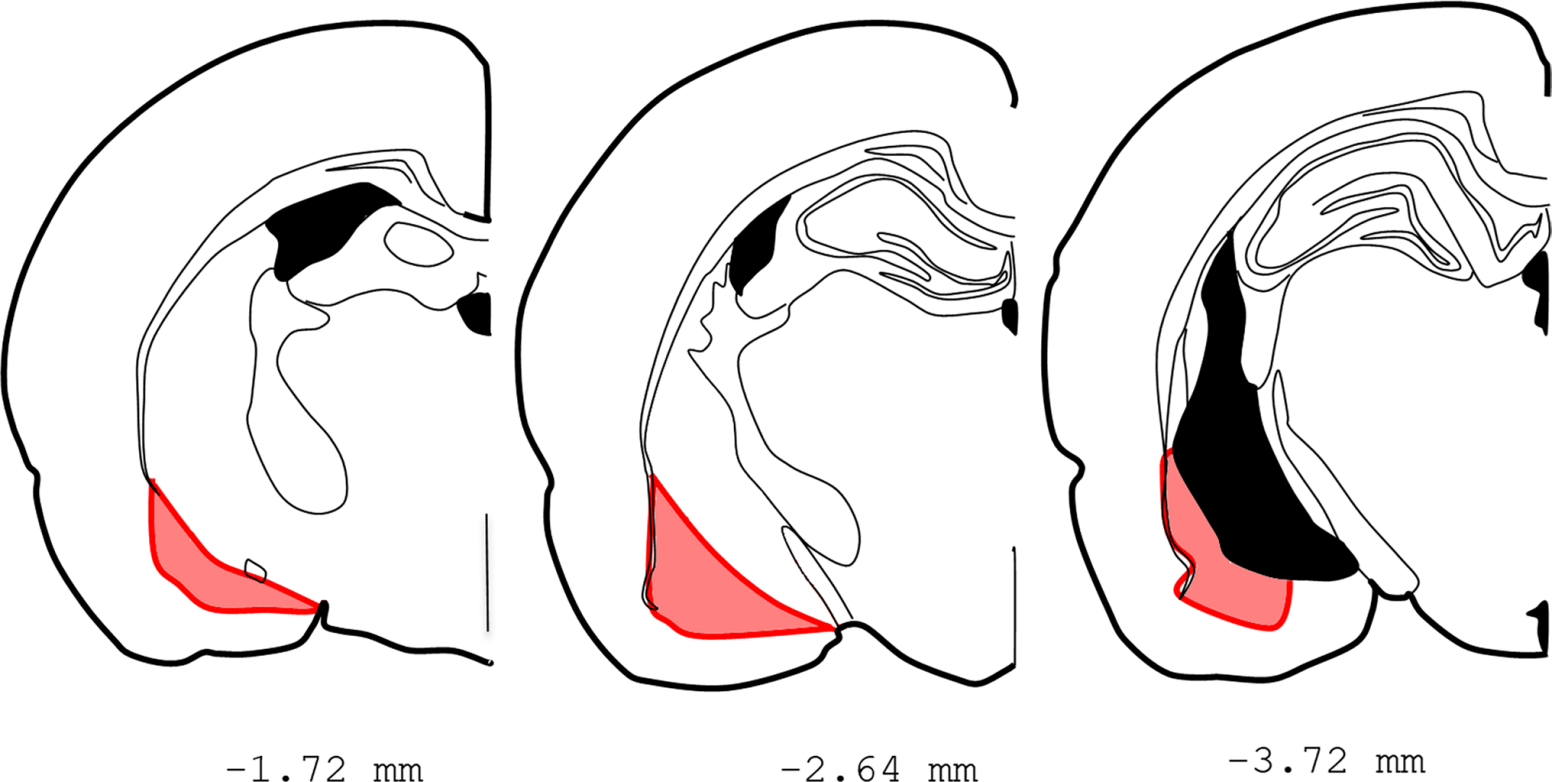
Graphical representation of the sections of the rat brain with the dissected area highlighted in red

For the dissection of the BCA, each brain was embedded in TissueTek (Sakura, 4583) and tempered at − 20 °C in a cryostat chamber (Microm, Cryostat HM 500O). After 1 h, slices about 300 μm thick were collected and dissected with sterile equipment. The BCA dissection was performed following Paxinos and Watson, 2013 (Fig. 2), and the tissue was kept in dry ice until it was stored at − 70 °C.

Samples were weighed and homogenised with a pellet pestle (Sigma-Aldrich, Z359971) in HEPES buffer (50 mM, pH 7.5, Sigma H3375) prepared in diethylpyrocarbonate (DEPC)-treated water, and containing sucrose (320 mM, Sigma S1888), protease inhibitors (Roche complete EDTA-free 11.873.580.001) and phosphatase inhibitors (Roche PhosSTOP 04.906.837.001) and sodium butyrate (20 mM, Sigma B5887). The homogenates were kept on ice for 10 min and then centrifuged for 10 min at 1000 g at 4 °C. The supernatant was separated into three different tubes: an aliquot equivalent to 3–4 mg of tissue (≤ 80 μL) in a tube with 800 μL of QIAzol (Qiagen 79,306) for RNA isolation; another aliquot in a tube with loading buffer for western blotting; and the rest into an empty tube for protein quantification and other experiments. All the aliquots were stored at − 70 °C.

### RT-qPCR analysis

The total RNA was extracted and precipitated using the chloroform, isopropanol and ethanol method (Chomczynski and Sacchi 1987) with glycogen as a carrier. The precipitate was dissolved in RNAs-free water, and the concentration and RNA integrity (as indexed by the RIN value) was assessed in a Bioanalyzer 2100 (Agilent). The RNA concentration in each sample was adjusted by adding RNAse-free water and, to avoid genomic DNA contamination, DNAse digestion was performed (DNAse I, Amplification Grade, Invitrogen) following the manufacturer’s instructions. Finally, the samples were retrotranscribed using a commercial kit (Biorad iScript™ cDNA Synthesis Kit), and the cDNA was diluted 1:10 in nuclease-free water. PCR assays were performed on a real-time PCR detection system (CFX9600, Biorad) with an SSO Advanced SYBR mix (Biorad) using the primers indicated in Table 1. We ran duplicates of all the samples along with a no-template control (NTC). We discarded the data of any assay with an unusual amplification or melt curve, or if the difference between duplicates was higher than one cycle. The relative expression of each gene was calculated as described in Pfaffl, 2001, using *Gapdh* as a reference gene and the reaction efficiencies were obtained using LinRegPCR software (Ruijter et al. 2009), and normalised with respect to the VhW1 group.

**Table 1:** List of primers pairs used for the RT-qPCR assays

Gene	Primer sequences (5′-3′)	
	Sense	Antisense
*Akt1*	CGCTTCTTTGCCAACATCGT	TCATCTTGATCAGGCGGTGT
*Akt2*	GGCACGCTTTTATGGAGCAG	ATCTCGTACATGACCACGCC
*Gapdh*	TCCCTGTTCTAGAGACAG	CCACTTTGTCACAAGAGA
*Gsk3a*	GCCCAACGTGTCCTACATCT	TTGGCGTCCCTAGTACCTTG
*Gsk3b*	CCGAGGAGAGCCCAATGTTT	CTTCGTCCAAGGATGTGCCT
*Igf1r*	ATCTCCGGTCTCTAAGGCCA	CCAGGTCTCTGTGGACGAAC
*Igf2r*	TCACAATCGAGGTGGACTGC	CACCCGGTGACAGACATTGA
*Insr*	GCTTCTGCCAAGACCTTCAC	TAGGACAGGGTCCCAGACAC
*Mtor*	GGTGGACGAGCTCTTTGTCA	AGGAGCCCTAACACTCGGAT
*Rp6kb1*	ACTGGAGCACCTCCATTCAC	GCTTGGACTTCTCCAGCATC
*Pdk1*	GAAGCAGTTCCTGGACTTCG	GCTTTGGATATACCAACTTTGTACC
*Pik3ca*	GAGCACAGCCAAGGAAACTC	TCTCCCCAGTACCATTCAGC
*Rptor*	CTTGGACTTGCTGGGACGAT	ATGAAGACAAGGAGTGGCCG
*Rictor*	CCGTCGCAGCAATCAAAGAC	CCCCCAATTCGATGAGCCAA
*Rps6*	CGTCTTGTTACTCCCCGTGT	GCCTACGTCTCTTGGCAATC
*Eif4ebp2*	TCCTGGCGCCTTAATGGAAG	AAGATGTGGCTGGACAGAGC

### Western blot

After the quantification of the protein concentration of the samples with the Bradford assay (Bradford 1976), the equivalent to 5 µg of protein was mixed with 6 × Laemli buffer and loaded in 10% Mini-PROTEAN® TGX Stain-Free™ Protein Gels (Bio-Rad Laboratories, Inc.). The proteins were resolved by SDS-PAGE electrophoresis, and the gels were exposed to UV light for 1 min in order to visualise and quantify the total migrated proteins (Ladner et al. 2004) with an Amersham Imager 600 (GE Healthcare). The proteins were transferred to PVDF membranes (Trans-Blot Turbo Mini 0.2 µm PVDF Transfer Packs, Bio-Rad Laboratories, Inc.), blocked for 1 h in EveryBlot blocking buffer (Bio-Rad Laboratories, Inc.) and incubated overnight at 4 °C in Tris-buffered saline with Tween (TBST: Tris 50 mM [pH 7.5], NaCl 150 mM, Tween20 0.1% v/v) and a primary antibody (1:3000 v/v Rictor (53A2) Rabbit mAb, Cell Signaling Technology Inc., ref 2114, 1:3000 Phospho-Rictor (Thr1135) (D30A3) Rabbit mAb, Cell Signaling Technology, Inc., ref 3806). After washing the unbound antibody with TBST, we incubated for 1 h the membranes in TBST with secondary antibody conjugated with horseradish peroxidase (ab6721, Abcam plc.). We washed again the membrane with TBST and measured peroxidase activity with a chemifluorescent substrate (Thermo Scientific™ Pierce™ ECL 2, PI80196) and the Amersham Imager 600. Then, when necessary, the antibodies were stripped for reprobing by incubating the membranes for 45 min at 50 °C in a stripping buffer (Tris 62.5 mM [pH 6.8], SDS 2% w/v, β-mercaptoethanol 0.8% v/v). The images were analysed with the software ImageJ (Schindelin et al. 2012) for densitometry analysis.

### Statistical analyses

The data obtained from the self-administration experiment was analysed with a mixed-model repeated-measures approach, and the within-subject correlations were modelled using the first-order autoregressive covariance structure. The analysis had sessions as a within-subjects factor (ten levels) and treatment (three levels: heroin_tissue, heroin_test and vehicle_tissue) and withdrawal (two levels: 1 day, 30 days) as between-subject factors.

For the analysis of the incubation of drug-seeking, we performed a *t*-test to compare the means of the active lever presses during the seeking tests of the HTest1 and HTest30 groups. We also carried out an ANCOVA with inactive lever presses as a covariate in order to control the influence of this variable.

For the analysis of the biochemical variables, we used a two-way ANOVA with two factors: treatment (Two levels: heroin and vehicle) and withdrawal (two levels: 1 day and 30 days). In the case of a significant interaction, a simple effect analysis with a Bonferroni correction was performed.

Prior to these tests, we checked for normality and homoscedasticity and applied log_10_, square root or reciprocal transformations. If the assumptions were still violated, a Kruskal–Wallis non-parametric test was performed instead of ANOVAs.

The statistical analyses were performed using SPSS 24 (IBM) and InVivoStat (Mockett Media). The graphs were designed using PRISM 6 (Graphpad software Inc.). The diagrams of the signalling pathways were created with BioRender.com.

## Results

### Heroin self-administration

**Fig. 3:**
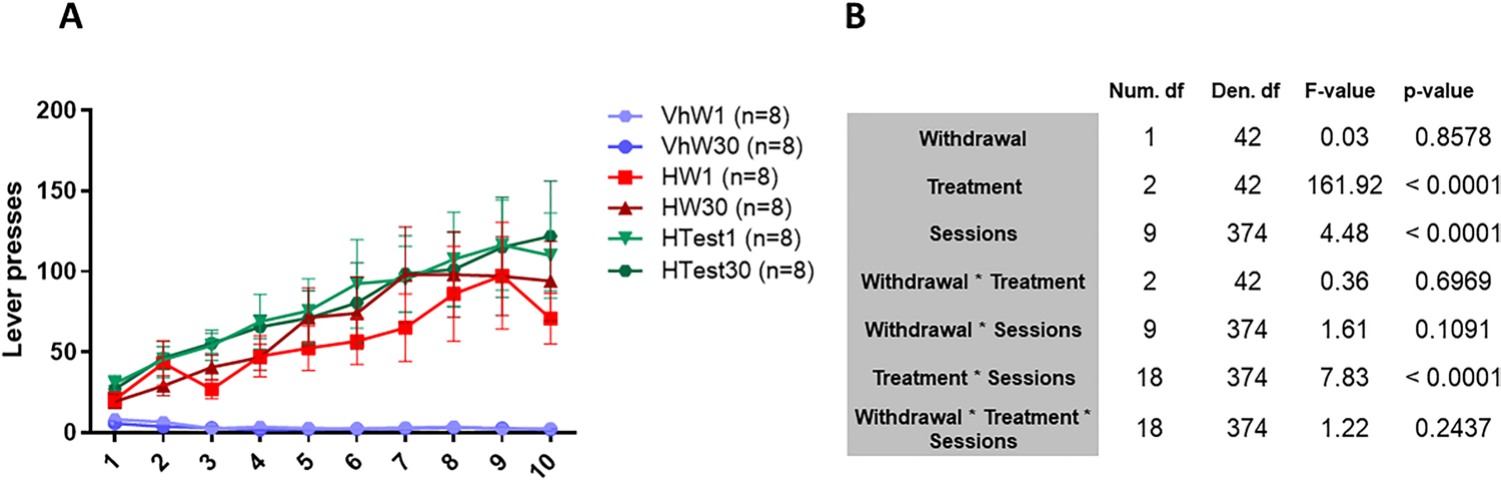
**A** Graphical representation of the self-administration data (VhW1, vehicle withdrawal 1 day; VhW30, vehicle withdrawal 30 days; HW1, heroin withdrawal 1 day; HW30, heroin withdrawal 30 days; HTest1, heroin withdrawal 1 day seeking test; HTest30, heroin withdrawal seeking test). **B** Table of overall tests of model effects for self-administration data

The rats of the groups that self-administered heroin, either the tissue collection or the test groups, acquired the lever press behaviour and, as the sessions went on, self-administered more heroin (Fig. 3A). There was an effect of the session factor (*F*_9,374_ = 4.48, *p* < 0.0001), indicating that the behaviour changed throughout the sessions. We also found an effect of the treatment factor (*F*_2,42_ = 161.92, *p* < 0.0001), and a session × treatment interaction (*F*_18,374_ = 7.83; *p* < 0.0001) (Fig. 3B). The post hoc analyses indicated that, as expected, the vehicle-treated animals displayed fewer responses in all the sessions than the groups that self-administered heroin, both in the tissue or the test group. There was a session in which the test group and the tissue groups behaved significantly different, but, as there was no overall difference, it was unlikely that this small effect could have affected our results.

### Incubation of heroin-seeking

**Fig. 4:**
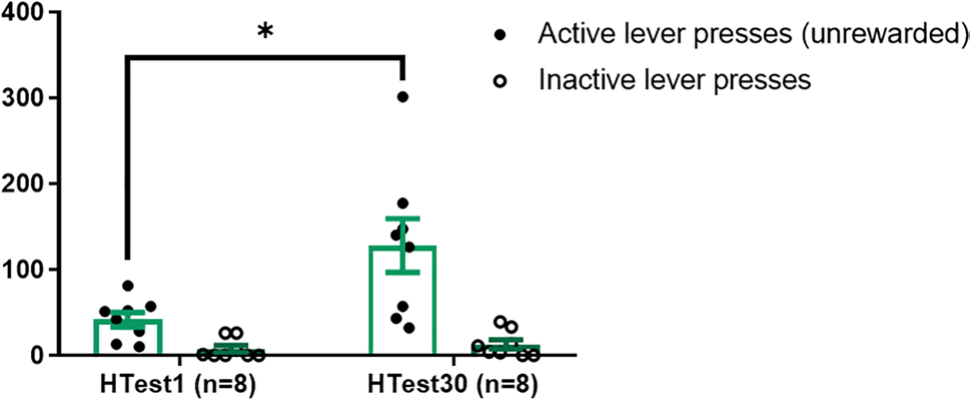
Results of the seeking tests evidencing the incubation of heroin-seeking. Mean and SEM of the previously active or inactive lever presses during the seeking tests performed after 1 or 30 days of forced withdrawal from heroin self-administration. The HTest30 group displayed more responses in the active lever during the seeking tests than the HTest1 group, even when accounting for the responses in the inactive lever (F1,13 = 5.732, *p* = 0.032, *η*2 = 0.44)

The data obtained in the seeking tests showed that our protocol induced incubation of seeking. Indeed, the rats in the long withdrawal group (HTest30) displayed more active lever presses than the rats in the short withdrawal group (HTest1) (t8.022 =  − 2.669, *p* = 0.028, *d* = 1.335), even when controlling the inactive lever presses as a covariate (*F*_1,13_ = 5.732, *p* = 0.032, *η*2 = 0.44) (Fig. 4).

### RNA integrity

All the samples had a RIN value of 7 or higher and, in most cases, the 260 nm/280 nm absorbance ratios were 1.8 or higher.

### Gene expression

**Fig. 5:**
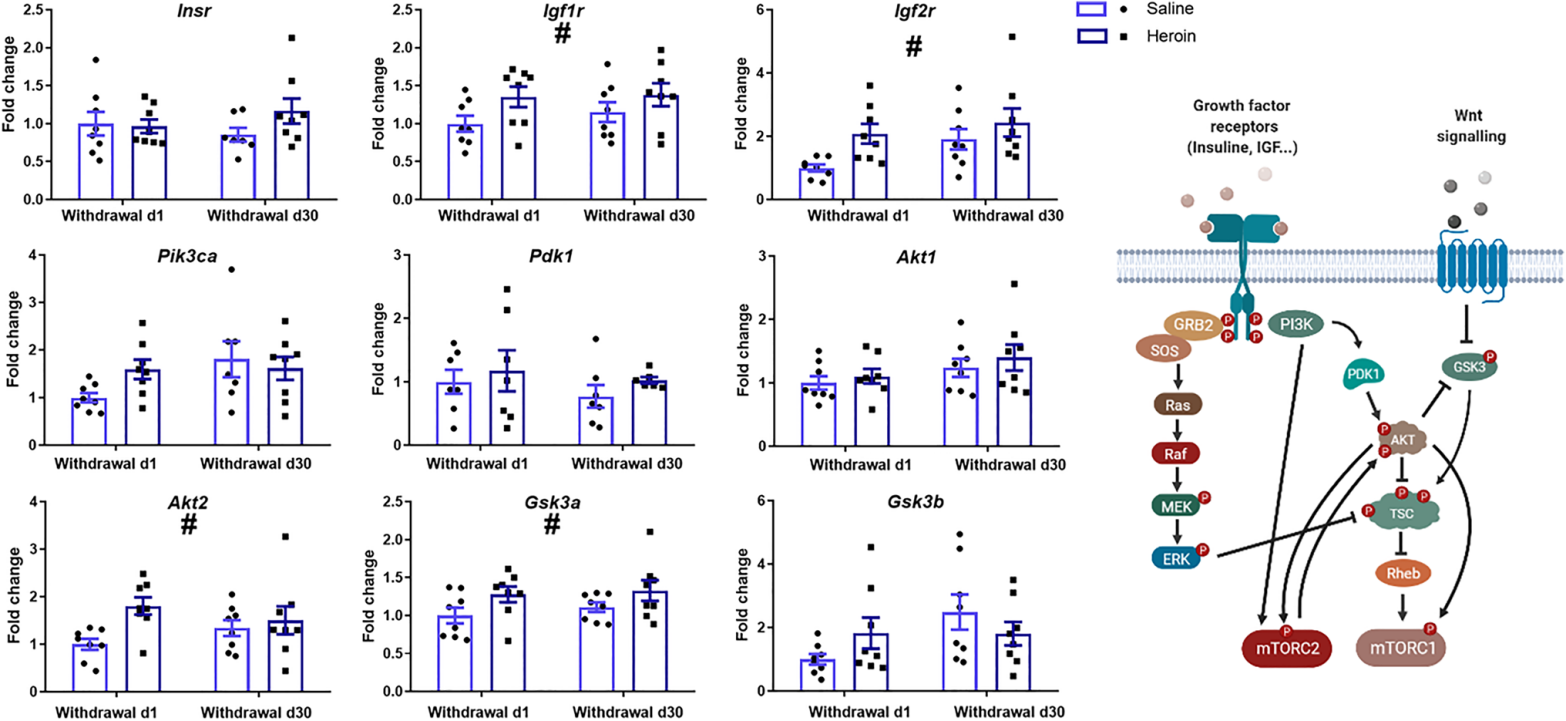
Mean and standard deviation of the relative expression of the genes coding several activators of the mTOR network studied, normalised to saline/withdrawal 1 (VhSA) values. The rats of the groups that self-administered heroin showed higher mRNA levels of *Igf1r* (F_1,28_ = 4.931, *p* = 0.035, *η*2 = 0.147), *Igf2r* (F_1,2_8 = 6.277, *p* = 0.018, *η*2 = 0.161), *Akt2* (F_1,28_ = 5.763, *p* = 0.023, *η*2 = 0.158) and *Gsk3a* (F_1,28_ = 5.556, *p* = 0.025, *η*2 = 0.162). ^#^Significant effect of the treatment factor (*p* < 0.05)

We found that the expression of several genes was increased in the BCA after heroin self-administration, and these effects were still evident after 30 days of withdrawal. The genes which showed significant treatment effects in the ANOVA were *Akt2* (*F*_1,28_ = 5.763, *p* = 0.023, *η*2 = 0.158), *Gsk3a* (*F*_1,28_ = 5.556, *p* = 0.025, *η*2 = 0.162), *Igf1r* (*F*_1,28_ = 4.931, *p* = 0.035, *η*2 = 0.147) and *Igf2r* (*F*_1,28_ = 6.277, *p* = 0.018, *η*2 = 0.161) (Fig. 5).

**Fig. 6:**
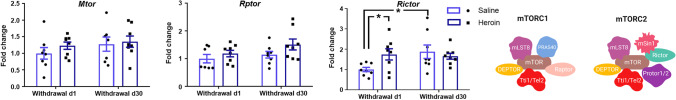
Mean and standard deviation of the relative expression of the genes coding for the same of the proteins that constitute the mTOR complexes, normalised to Saline/Withdrawal 1 (VhSA) values. The rats that self-administered heroin showed higher mRNA levels of Rictor than their saline-treated controls after 1 day of withdrawal (*F*_1,28_ = 5.094, *p* = 0.032, *η*2 = 0.182). Also, among the groups that self-administered saline solution, the rats that underwent 30 days of withdrawal showed higher *Rictor* mRNA levels than the rats that only had one day of withdrawal (*F*_1,28_ = 7.104, *p* = 0.013, *η*2 = 0.254). *Significant effect of the simple effect analyses (*p* < 0.05)

**Fig. 7:**
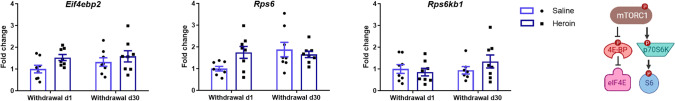
Mean and standard deviation of the relative expression of the genes coding for some of the effectors of the mTOR complexes, normalised to saline/withdrawal 1 (VhSA) values

We also found a significant interaction between the treatment and withdrawal factors concerning the expression of *Rictor* in the BCA (*F*_1,28_ = 4.293, *p* = 0.021, *η*2 = 0.118). The simple effects analysis showed that the animals had higher levels of *Rictor* expression after heroin self-administration compared to their controls (*F*_1,28_ = 5.094, *p* = 0.032, **η**2 = 0.182) and that there was an increase after the withdrawal in the expression of *Rictor* in the BLA of the rats that self-administered vehicle (*F*_1,28_ = 7.104, *p* = 0.013, *η*2 = 0.254), while the rats that self-administered heroin had already elevated levels before withdrawal (Fig. 6). In addition, *Eif4ebp2* expression also seemed to increase, but this surge did not reach the traditional threshold for statistical significance (*F*_1,28_ = 4.004, *p* = 0.055, *η*2 = 0.12) (Fig. 7).

### Protein levels and phosphorylation

Although we did not find significant changes in the levels of Rictor or phospho-Rictor (thr1135) corrected for the total protein loaded, there was a significant effect of the Treatment in the phosphor-Rictor (th1135)/Rictor ratio (*F*_1,22_ = 5.247, *p* = 0.032, *η*2 = 0.155). It must be noted that even if this effect seems to be specific to the HW30 group, the treatment × withdrawal interaction was not significant (*F*_*1*,22_ = 3.047, *p* = 0.095).

## Discussion

In this study, we analysed the gene expression of several elements related to the mTOR network in Lewis rats that had extended access to heroin or saline, either 1 or 30 days after the last self-administration session. We also confirmed in a separate group of rats the existence of incubation of heroin-seeking using this behavioural protocol (Roura-Martínez et al. 2020b).

Our main finding was an increase in the expression of *Akt2*, *Gsk3a*, *Igf1r* and *Igf2r* in the rats that self-administered heroin compared to their vehicle controls, a change still evident after one month of withdrawal (Fig. 5). These genes are closely related within the mTOR network (Ucha et al. 2020). The activation of Insulin-like growth factor receptors promotes the phosphorylation of PI3K, which in turn mediates the phosphorylation of Akt (Alessi et al. 1996; Dudek 1997), and GSK3 inhibition is regulated by Akt kinase activity (Cross et al. 1995). As shown in Fig. 5, the effects of this signalling cascade converge in the tuberous sclerosis complex 2 (TSC2) (Inoki et al. 2002, 2006) and, by inhibition of this complex, it induces the activation of the mTORC1 through Rheb activity (Long et al. 2005). In addition, the mTORC2 is an upstream regulator of Akt (Sarbassov et al. 2005) and, at the same time, mTORC2-dependent Akt phosphorylation is negatively modulated by one of the effectors of the mTORC1 (p70 ribosomal S6 kinase 1, Fig. 7: *Rps6kb1*) (Julien et al. 2010). This negative feedback shows the intricate interplay between the complexes of the network.

Although there are no previous reports of overregulation of this pathway in the BLA after opiate self-administration, there are reports of similar effects in vitro or in other brain areas. For example, plasma levels of IGF1 were transiently increased after intracerebroventricular administration of morphine, another opioid (Hashiguchi et al. 1996). In vitro studies show that opioid stimulation elicits an increase in Akt activation (Polakiewicz et al. 1998; Li et al. 2003), but the studies in vivo show a more complex regulation. Although acute morphine administration produced an increase in Akt activity in the NAcc (but not in the striatum), chronic administration had the opposite effect, although total protein levels remained unchanged (Muller and Unterwald 2004).

Similarly, in other in vivo studies, chronic morphine produced a decrease in Akt and GSK3 activity in the VTA, while PI3K and PDK1 remained unchanged. These changes were related to a decrease in the rewarding properties of morphine and are supposed to be involved in the tolerance to opioids (Russo et al. 2007; Mazei-Robison et al. 2011). Whether our findings in the BLA are related or not to tolerance or any other addiction-related phenomenon will require further testing.

GSK3 activation in the BLA is increased after exposure to drug-associated cues and is involved in the association of incentive value to these cues (Wu et al. 2011). In our study, we found an increased expression of *Gsk3a* in the same area. Being GSK3 an upstream inhibitor of the mTORC1, the fact that we found also higher expression of some upstream activators of this complex can be confusing. This conflict is also reflected in other studies. For example, the reconsolidation of drug-related memories can be prevented by systemic inhibition of either the mTORC1 (Barak et al. 2013) or GSK3 (Shi et al. 2014), and by GSK3 inhibition in the BLA (Wu et al. 2011). This could mean that GSK3 could be acting through another of its more than 100 known substrates (Beurel et al. 2015) and, if this were true, the identification of this effector would be of great interest (see Ucha et al., (2020) for further discussion on this issue).

We also found an interesting trend regarding *Eif4ebp2* expression, which tended to be increased in the rats that self-administered heroin (*p* = 0.055). This is interesting because, in a previous study, we found a similar effect in rats that had self-administered morphine, and this effect persisted after 15 extinction sessions (Ucha et al. 2019). *Eif4ebp2* encodes one of the effectors of the mTORC1 (Shimobayashi and Hall 2014) which, when not phosphorylated, inhibits cap-dependent mRNA translation by binding to the EIF4E (Richter and Sonenberg 2005). The activation of EIF4E through mTORC1 is also mediated by Akt (Wendel et al. 2004), which showed elevated expression, as discussed before. In another study, the levels of EIF4EBP (total and phospho-EIf4EBP) were increased in the VTA, but not in the accumbens, after chronic morphine administration (Mazei-Robison et al. 2011), and there is evidence as well of opioid-stimulated phosphorylation of EIF4EBP1 and EIF4EBP2 in vitro (Polakiewicz et al. 1998).

**Fig. 8:**
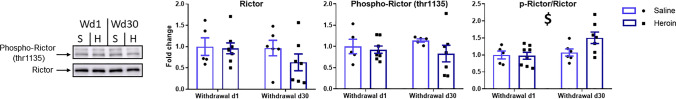
Representative western blot images and densitometry bar graphs normalised to total protein loaded (Rictor and Phospho-Rictor (thr1135) or to Rictor levels (p-Rictor/Rictor)). The rats that self-administered heroin showed higher phosphorylation rates at thr1135 than their saline-treated controls (*F*_1,_22 = 5.247, *p* = 0.032, *η*2 = 0.155). ^$^Significant effect of the treatment factor (*p* < 0.05)

We also found a surge in the expression of Rictor, one of the proteins of the mTORC2, in the rats that self-administered heroin compared to their controls after 1 day of withdrawal. In this case, after the long withdrawal, both heroin and vehicle-treated animals had a similar increase in Rictor expression. The change in the rats of the control groups may be a result of the experimental manipulations or the natural course of the regulation of this gene. This notwithstanding, the increased levels of Rictor transcripts after heroin self-administration suggested an involvement of the mTORC2 in the changes we have seen in the BLA of heroin treated animals. Apart from the classic PI3K/Akt pathway mentioned before, there are several activators of Akt, and mTORC2 is one of them (Sarbassov et al. 2005). Following this lead, we analysed the levels of Rictor protein and its inactivation through phosphorylation at thr1135, which is known to downregulate mTORC2-directed Akt phosphorylation and signalling through an unknown mechanism independent of mTORC2 formation, location or kinase activity (Julien et al. 2010). Although there were no significant changes in the levels of Rictor or the phosphorylated protein, we did find an increase in phosphor-Rictor/Rictor ratio in the rats that self-administered heroin, an effect apparently specific to the protracted withdrawal group. This phosphorylation is supposed to be mediated by the p70 ribosomal S6 kinase 1, one of the effectors of the mTORC1 (Julien et al. 2010). Interestingly, the expression pattern of this protein (see *Rps6kb1*, Fig. 7) is similar to the phosphorylation ratio observed in Rictor (Fig. 8), although the modulation pattern observed was not significant. This phosphorylation is also regulated by PTEN phosphatase activity through unknown mechanisms. Moreover, PTEN activity also inhibits mTORC2 complex formation (Bhattacharya et al. 2016). The study of the involvement of this phosphatase in the mechanisms of reward and addiction could be of great interest since there is previous evidence of regulation of complex behaviours by amygdalar PTEN (Sánchez-Puelles et al. 2019)**.**

The protein levels of Rictor did not match with the changes we found in the RNA transcripts. The increased Rictor transcription after 1 day of withdrawal was not evident at the level of translated protein, and we found an increase in the ratio of phosphorylated Rictor which may be specific to the rats that underwent 30 days of withdrawal. This would suggest a dampened activity of the mTORC2. The impact of this regulation on the mechanisms of opiate reward and its expression is yet to be explored. Of note, in another study, the changes in opioid-related Akt activation in the VTA were studied by evaluating the phosphorylation of substrates of both mTOR complexes. The authors found that both of them were affected, but the related changes in behaviour and VTA cell physiology were dependent on a reduction in mTORC2 activity only (Mazei-Robison et al. 2011). Although the studies differ in the area studied (VTA and BCA) and paradigm (chronic morphine and heroin self-administration/forced withdrawal), the results suggest a reduction of the mTORC2 activity related to opioid administration and its putative role in the mechanisms behind opiate use disorders should be further studied.

## Conclusions

This study advances our understanding of the alterations of the mTOR network after heroin self-administration under conditions that promote the development of compulsive intake, escalation and incubation of seeking. However, caution is in order when trying to directly connect these changes with the actual incubation phenomenon as most of our results were not dependent on the incubation period.

One of the limitations of this study lies in the timing of the late withdrawal groups (HW30 and HTest30). Other studies have shown that the peak of “incubated” heroin-seeking in withdrawn rats occurs at around 6 days (Shalev et al. 2001). It is then possible that, since our rats were euthanized weeks after the supposed incubation peak, some of the molecular changes involved could have been missed by our design. Nevertheless, we found that opiate exposure and withdrawal increase the expression of several elements of this signalling pathway, and the effects observed for Rictor mRNA and phosphorylation suggest that the mTORC2 activity could be affected after heroin withdrawal. These results open new avenues for research into pharmacological manipulations of these elements as potential therapeutic targets for opioid use disorders.
